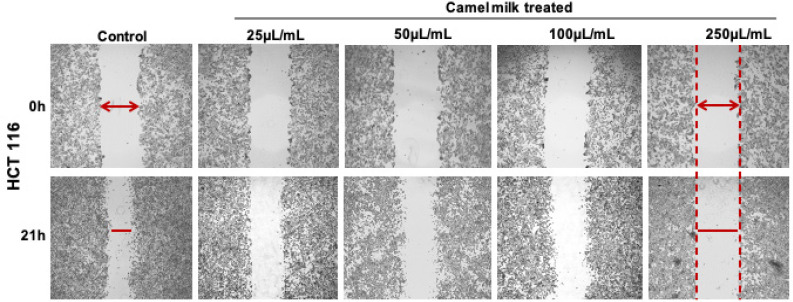# Erratum

**DOI:** 10.31557/APJCP.2020.21.5.1495

**Published:** 2020-05

**Anticancer Activity of Camel Milk via Induction of Autophagic Death in Human Colorectal and Breast Cancer Cells. Asian Pac J Cancer Prev. 2018; 19(12): 3501-3509. doi: 10.31557/APJCP.2018.19.12.3501. Roopesh Krishnankutty et al.**

*Figure Correction*

In the published article “Roopesh Krishnankutty et al. Anticancer Activity of Camel Milk via Induction of Autophagic Death in Human Colorectal and Breast Cancer Cells”. Asian Pac J Cancer Prev. 2018;19(12):3501-3509”, the images in Figure 3a for dose points 25, 50 and 100µL/mL were wrongly presented as same (indicated in red boxes). This mistake was a technical error, the image for one dose point was erroneously copied to subsequent two dose points. The mistake has now been corrected by replacing them with original images corresponding to the respective doses and can be found in the corrected Figure 3a as below. The authors apologize for this mistake and any inconvenience caused by it. 

**Figure:**
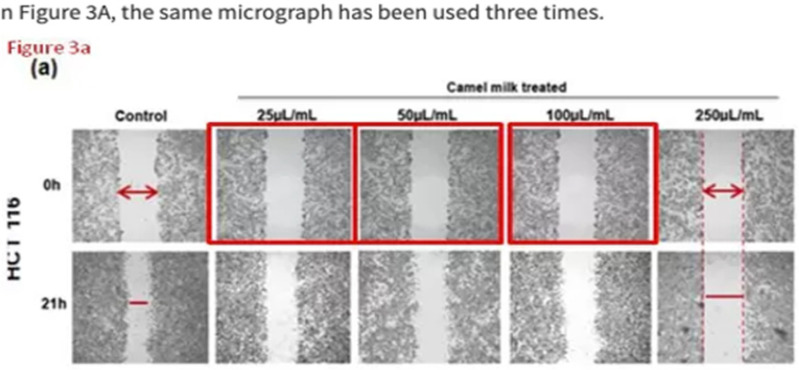


*The corrected Figure 3a as below:*

**Figure:**